# Impact of Traumatic Material on Professionals in Analytical and Secondary Investigative Roles Working in Criminal Justice Settings: a Qualitative Approach

**DOI:** 10.1007/s11896-022-09532-8

**Published:** 2022-07-21

**Authors:** Fazeelat Duran, Jessica Woodhams

**Affiliations:** School of Psychology, University of Birmingham, 52 Pritchatts road, Edgbaston, Birmingham, B15 2TT UK

**Keywords:** Traumatic material, Distressing material, Analysts, Secondary investigators, Psychological distress, Mental health

## Abstract

Professionals in analytical and secondary investigative roles are exposed to violent material on a daily basis with full immersion in the details of serious offenses. However, there is limited evidence of the impact of this on their mental health. Therefore, this research aims to explore the impact of traumatic material on the mental health of these professionals in police and law enforcement and the strategies they employ to cope with the nature of their work. Forty semi-structured interviews were conducted with these professionals from UK, Canada, and Europe. Five key themes were identified: “Analyzing material,” “Negative Influences,” “Coping Strategies,” “Additional Risk Factors,” and “Protective Factors.” From the findings, it is evident that these professionals are at significant risk of developing secondary traumatic stress, burnout, and sleep problems. The constant exposure to this material negatively influenced their feelings about their home and social lives. The implications of these findings and avenues for providing a supportive working environment are discussed.

Policing is widely accepted as one of the stressful and traumatic professions (Greshon et al. 2009). Concerns are being raised on an international scale regarding the mental ill-health of staff in our police, security, and justice organizations as this could negatively impact their ability to protect the nation and fundamental human rights to justice and security (Duxbury and Higgins 2012). The exposure of frontline police officers and investigators to other people’s trauma at work (henceforth traumatic material) and its negative impact on them is well-researched (Cartwright and Roach 2020; Duran et al. 2019; Hurrell et al. 2018; Tehrani 2018). However, little-known is that much of the work of police, law enforcement, and justice organizations is completed by staff in “supporting roles” who are rarely in the public eye but whose work is just as important for our rights to security and justice. These professionals include crime analysts, behavioral investigative advisors, intelligence analysts and officers, digital forensics analysts, police and court translators, and researchers, who are regularly exposed to traumatic experiences of other people indirectly as a part of their daily work via auditory and/or visual means. Their job role is to provide analysis and intelligence and support to investigations and prosecutions for the most serious of crimes. Despite the significant trauma to which they are exposed and the importance of their role in security and justice outcomes, they are rarely the subject of academic research (Duran and Woodhams 2022; Perez et al. 2010; Tehrani 2018; Starcher and Stolzenberg 2020; Wortley et al. 2014).

A key aspect of the work of law enforcement back-office staff is the volume and continuous exposure to new traumatic material. Their daily tasks involve full immersion in the details of murder, homicide, arson, sexual violence, child pornography, abduction, torture, genocide, and many more. Information about trauma is received by these workers via auditory and visual means (e.g., watching footage of abuse, watching a victim’s video interview), solely visual (reading detailed accounts of abuse), or solely auditory (e.g., listening to torture). Some staff analyze the behavior displayed in great detail to produce an analytical product (e.g., a crime linkage report), some classify images of child sexual abuse, and some translate accounts of abuse from one language to another, whereas others have to put themselves in the shoes of the victim or offender in producing behavioral investigative advice or taking on the persona of an offender (Brady 2017; Lavis 2012). Although these professionals are exposed more and in greater depth to significant trauma than first responders, yet they may be less resilient (Garborino et al. 2013). As a result of their regular exposure to traumatic material, limited studies with *some* of these analytical and secondary investigators have found they are at increased risk of secondary traumatic stress and burnout (Brady 2017; Fansher et al. 2019; Kunst et al. 2017; Lavis 2012; Perez et al. 2010; Startcher and Stolzenberg 2020; Wortley et al. 2014). However, these studies have been conducted with a single group of police or law enforcement staff in supporting roles often limited to one unit and, in all cases, to one country (Brady 2017; Fansher et al. 2019; Perron and Hiltz 2006).

## Traumatic Material and Mental Health

The ramifications of work-related indirect exposure to traumatized material have been conceptualized using several terms in literature, namely, vicarious traumatization (McCann and Pearlman 1990), compassion fatigue, PTSD-like symptoms, and secondary traumatic stress (Figley 1995). These terms are used interchangeably in literature; however, there are subtle differences between them (Jenkin and Baird 2002). Previous literature conducted with some of the crime justice workers indicated that analysts or secondary investigators who are indirectly exposed to other people’s trauma are at risk of developing secondary traumatic stress (STS) and burnout. For example, studies with forensic interviewers (Perron and Hiltz 2006), social workers (Bride et al. 2007), sexual assault advocates (Baird and Jenkins 2003), child care workers (Sprang et al. 2011), child welfare workers (Salloum et al. 2015), and child abuse investigators (Brady 2017; Hurrell et al. 2018; Wortley et al. 2014) indicated these professionals show secondary trauma symptoms due to the exposure to traumatic material, while burnout was due to heavy caseloads and shorter deadlines that cause greater exposure to traumatic material in a limited amount of time (Graef and Hill 2000; Fansher et al. 2019). Indeed, some studies have shown that having a heavy workload does not influence STS; however, the job-related demands like time spent working with disturbing material was significantly associated with high STS (Cieslak et al. 2007; Perez et al. 2010).

## Traumatic Material and Coping Mechanism

There are a few studies with these group of professionals that discuss the coping mechanisms individuals employ when faced with trauma. For instance, in a study with online child exploitation investigators, peer support and family support emerged as a crucial coping strategy. However, they also used cognitive avoidance to avoid distressing material and a detachment-based strategy where they shut-off emotions that affect their well-being (Lee et al. 2020). It was noted that humor was another coping mechanism where professionals in area of sexual violence often used gallows and light-hearted humor that reduced their work-related stress causing burnout and improved their relationships with co-workers. Similar strategies have been reported by frontline officers (Clark et al. 2015; Sollie et al. 2017). Furthermore, Slack (2020) discussed management strategies like partaking in activities, namely, exercise, prayer, mindfulness, and meditation. Given growing evidence of the potentially harmful effects of working with traumatic material in a secondary investigative/analytical role, there is a need to identify coping mechanisms used by such professionals to minimize the risk of mental-ill health that can be shared with others.

In summary, many professionals in the criminal justice system work in a secondary investigative, analytical, and back-office roles. Their work is key to the functioning of our criminal justice systems. While there is some existing research that demonstrates the deleterious effects of being exposed, daily, to traumatic material in these roles, these studies have tended to have a narrow focus, as explained above. In contrast, in this paper, we recruited participants from different units who all support investigative work but in varying roles, working with auditory, visual, or visual-auditory material, from a range of countries. As well as seeking to understand from them how they work with traumatic material, we explored the impact that this has on them and the coping mechanisms they utilize.

## Method

### Participants and Procedure

This study received ethical approval from the University of Birmingham and approval for the study was given by the participants’ employing organizations. The participants were recruited from different international and national police and law enforcement organizations via their employers. Purposive sampling strategy was used to recruit staff in police and law enforcement organizations who were exposed to traumatic material on a daily basis as a part of their job. All were working in analytical, intelligence-development, and secondary investigative roles, i.e., they were working with secondary data (data that has been collected from the primary source by a frontline officer). Their job roles were reasonably equivalent across jurisdictions. Those participants who were working “in the field” or who were front line were excluded from this study. In total, forty professionals from the UK, Europe (Belgium, Spain, Netherland), and Canada, aged 23–55 years, participated. Twelve were uniformed officers and twenty-eight were civilians. Twenty-nine referred to themselves as female and eleven as male. Their length of operational service ranged from 7 months to 27 years.

They were indirectly exposed to a wide range of traumatic material on a day-to-day basis in different forms as a part of their job. The types of material they were exposed to were sexual assault, rape, murder, homicide, arson, child exploitation, torture, abduction, acts of terrorism, extortion, human trafficking, exhibitionism, rival gang fights, burglary, genocide, and any offense that had a violent element. The mode through which they received the information was case- and unit-dependent, that is, in some units they were exposed to written material, like police statements and victims’ reports, while in other units they were exposed to CCTV videos/images, online videos, victims’ video interviews, and autopsy images. In addition, interviewees mentioned that the “duration of exposure” depended on their working hours that is 35 to 50 hours a week excluding overtime.

All participants gave informed consent prior to taking part in a telephone interview and the information sheets were tailored for each organization to include the details of each specific well-being service and sources of support outside work so that interviewees could seek help immediately, if required. Before the interview, consent was obtained for audio recording the interview. The interviews were held for 30–55 min (*M* = 43 min). Each interviewee was given a 2-week cooling-off period post-interview to withdraw from the study if they wished and audio files were transcribed, replacing the names with pseudonyms. Data saturation was reached after thirty-one interviews.

### Materials

A semi-structured audio interview was conducted with each participant on an individual basis. At the start of the interview, demographic information was collected from interviewees, namely, age, gender, marital status, job title, and number of years employed in the organization. Following that, open-ended questions were asked regarding the type of distressing material they worked with and the frequency of exposure to that material. They were asked what cognitive processes they might use when working with or analyzing the material, the positive and negative effects that their work might be having on them (if any), and how they coped with the nature of their work.

### Analysis

The transcriptions were analyzed using template analysis because a priori themes were identified in advance of interviews and coding which formed the basic structure of the semi-structured interview (Kings and Brooks 2018). Template analysis is a flexible technique that allows the researcher to tailor the approach according to the requirements of the project. For example, it allows for a priori themes as well as themes that are identified during the data coding process. A priori themes were already present in our interview schedule (i.e., some themes were present due to the questions we asked—see above) that was informed by previous literature and our pilot study (Lavis 2012). The initial template generated by the first author was applied to the first eleven transcripts with sections of text being labeled with the theme they represented. Following initial analysis, this template was refined by both authors (FD and JW) to ensure codes accurately captured the themes within the interviews and a final template was produced, which was applied to the full data set (Brooks and King 2014). Twenty-five percent of the transcribed data was coded and analyzed by an experienced template analysis coder to check consistency in themes. Finally, the authors examined the relationships between different themes. A part of our findings related to COVID-19 and how changes to working practices were experienced by staff in these roles. Given the need to disseminate this in a timely way, discussion of any such matters was extracted from the interviews and analyzed separately prior to the full analysis of the interviews, which is presented here (please see Duran and Woodhams 2022).

## Results

From forty interviews, five main themes were identified and labeled as “Analyzing material,” “Negative Influences,” “Coping strategies,” “Additional Risk Factors,” and “Protective Factors.” These themes were organized in hierarchical groups where the main theme served as an umbrella theme encompassing more specific themes. The final template is presented in Table 1.

**Table 1:** Themes from the template analysis

Key theme	Main theme	Sub-theme
Analyzing the material	Perspective taking when analyzing the material	From victim’s perspective; offender’s perspective; observer/analyst and victim’s perspective; not visualizing material
Negative influences	Perceptions following exposure to material	Feelings about the world (e.g., how uncertain the world is; a thin line between what is happening and could happen) Feelings about home-life (e.g., worried about loved ones) Feelings outside home (e.g., mindful of surroundings; having limited friends)
	Behavioral impact of traumatic material	Avoidance of situations (e.g., similar to the circumstances of crimes that they have read about or watched) Precautionary behavior (e.g., locking doors, windows, and cars or not letting their kids have a sleepover) Hyper vigilance (e.g., more wary and suspicious about their own and others’ safety)
	Emotional reaction to the material	No emotional reaction Negative emotions (e.g., sad, angry, disgusted) Gratitude for being safe in unsafe world
	Sleep problems	Poor sleep/insomnia; recurring thoughts; nightmares; no sleep problems
Coping strategies	Coping strategies when working with material (in situ)	Taking breaks from material Detachment-based strategy (e.g., reading like a story) Acceptance (e.g., part of job; accepting good and bad people in the world) Desensitized (due to excessive exposure) Communication (with peers) Awareness and preparedness (forewarned by colleagues about the challenging nature of a case) Avoidance of stimuli (similar to personal circumstances)
	Coping strategies when away from material	Compartmentalization (consciously not blending home and work life) Thought suppression Safety behaviors (e.g., avoiding to watch crime videos or novels, stopped running) Support (from employer, peers, and significant others) Relaxing activities (e.g., exercise, baking, music)
Protective factors	Personality	Optimistic
	Motivators	Intellectually stimulating Desire to help people Nice supportive environment
Additional risk factors	Pressure of workload	Focus on quantity not quality of work Inadequate staffing Insufficient time to access support
	Organizational culture	No other coping strategies (except communicating to peers); paying lip-service to well-being; concerned about reputation; social desirable responses in psychological assessment; neglected via employers; reactive not preventative support; management having limited knowledge; no training on working with this distressing material; lack of career development; lack of confidentiality; mental health stigma
	Consequences of additional risk factors	Feeling isolated; intrusive thoughts about work deadlines; poor sleep; decreased productivity; have to work extra hours (to meet deadlines); insufficient time for well-being services; unable to take leave (due to staff shortage); intentions to leave the job; seeking private mental health support (to avoid being stigmatized)

### Analyzing the Material

In their interviews, the analysts talked about engaging in “perspective-taking” when working with the material to understand victims’ or offenders’ behavior through their own senses, specifically via their minds’ eyes. Some of them (*n* = 8) visualized the material they were reading from the “victim’s perspective” because the written material was victim-led “I read the material from the victim’s perspective as the material is written from the victims’ perspective” (Pt. 31). Others (*n* = 14) perceived the material from both the “victims’ and analysts’ perspective.” They talked about taking both perspectives to understand the case by bringing different parts of information together. Two interviewees reported taking the “offender’s perspective” as they had to identify patterns in the offenders’ behavior. Finally, for three of the interviewees, the perspective they adopted was “gender-based.” For example, a male analyst would visualize the material from a male perspective irrespective of whether the male was the offender or the victim. However, a few of them (*n* = 7) mentioned that they do not visualize the material from mind’s eye at all. “I don’t visualize when working with the material” (Pt. 11).

### Negative Influences

This theme summarizes the negative impact of traumatic exposure on these professionals’ “Perceptions Following Exposure to Traumatic Material.” Furthermore, being exposed to other people’s trauma daily was influencing their “behavior,” “emotions,” and “sleep.” The sub-themes below mirror reported symptoms of secondary trauma stress, burnout, and sleep problems.

#### Perceptions Following Exposure to Traumatic Material

All of the interviewees mentioned experiencing changes in their perceptions following exposure the traumatic material due to the nature of their work. They mentioned how uncertain the world was and that there was a thin line between normal life and any traumatic incident that could happen in the world. Hence, they were “more aware of things” that could happen in the world; therefore, they felt working in this role was an “eye-opener” to them that showed the reality of the world. “This role has made me more aware of my surroundings and types of crime that happen in the world” (Pt. 15)*.* As a result, affecting their decision-making*.* Similarly, some of them (*n* = 16) mentioned that “crime seems to be never ending” in the world. “It seems the crime will never end in this world” (Pt. 24).

Their “feelings about home-life” were influenced because all participants reported being “worried about their loved ones” as they perceived certain environments to be risky. “I’m more worried about my sister if she would say I’m going for a walk in the night on a quiet road” (Pt. 4). In consequence, this perception was making them “more wary and cautious” than the average person.

In addition, when outside home, most of them (*n* = 25) were “mindful of their surroundings” and they had “limited friends” as they were cautious and cynical in making new friends and suspicious speaking to people outside their work. “I’m more socially aware of my surroundings … now, I’m more cautious and don’t make new friends easily” (Pt. 21). This suggested that they were more cynical in their attitudes toward other people and the world, and this belief was similar to burnout and STS symptoms observed in previous research (Bride et al. 2007; Maslach and Leiter 2008).

#### Behavioral Impact of Traumatic Material

More than three-quarters of the participants (*n* = 28) spoke about changes in their behavior as a result of exposure to traumatic material. Almost all of them mentioned that they have developed precautionary behaviors. Some behavior was to avoid intrusive thoughts. For example, they were “avoiding situations” that were similar to the circumstances of crimes that they have read about or watched. For example, they avoided walking on quiet and/or dark roads or alleys in the night-time. “When it is dark and I’m walking back from work to home, I avoid dark and quiet roads” (Pt. 1). In addition, a couple of them mentioned “reduced intimacy” with their partner because they would have intrusive thoughts during intimate moments, and four of them mentioned that they were avoiding men and not dating them because in most of the serious crime incidents, men were the offenders. “I’m a man hater, I feel if I date a man he would behave like one of the offenders from the cases I’ve worked on” (Pt. 23).

Other “precautionary behaviors” included locking doors, windows, and cars or not letting their kids have a sleepover. “How can I leave my children at someone’s place for a sleepover, I think I’m more skeptical than a normal parent would be” (Pt. 28). In previous studies, precautionary behaviors have been linked to reducing resilience and causing psychological distress like secondary trauma (Perez et al. 2010).

Some of the participants (*n* = 28) stated being “hyper vigilant” to threat cues, which is one of the main PTSD-type reactions (DSM-5). They were “more wary and suspicious” when in social gatherings and would look for potential threats and identify vulnerabilities. “When I was driving, I saw a man holding a young girl’s hand and I was hoping that she was not in danger but I could not do much about it” (Pt. 18). Similarly, several of them (*n* = 26) were cautious about their own safety. For example, they avoided opening the door to strangers and checked their car doors, house doors, and windows were locked multiple times. “I’m more cautious of being on 1–1 situation with a stranger, I think like hopefully it is ok” (Pt. 24). “I don’t leave a charger cord on my bedside, I think the burglar would strangle me with that” (Pt. 35).

#### Emotional Reaction to the Material

In terms of emotional reactions to the material, one-quarter of the participants (*n* = 8) stated they felt “no emotions” at all because they considered this material a part of their job. “I don’t show any emotions at all… I don’t know why…” (Pt. 31). Some of the more experienced participants (*n* = 7) disclosed that the material might have affected them at one time but they were “not aware this effect” now; hence, they were “not experiencing any emotions.” “It’s like I’m working with this material for long time and I’m not sure if it is affecting me or not, perhaps I’ve developed resilience to it” (Pt. 14). However, the majority of the interviewees (*n* = 19) spoke about getting “angry,” “frustrated,” “upset,” and “sad” when reading or watching the traumatic material. “It is not easy to watch the victims’ interviews, it upsets you and sometimes makes me angry” (Pt. 8). At times, there is an “emotional meltdown” when the trauma of another person was shown vividly in the written reports or victim interviews, or if, in some way, it related to their personal lives. “At times the victim’s report is written in detail that I’m at a verge of, emotional meltdown” (Pt. 27). For example, the participants “felt disgusted” when they read and/or watched material related to child exploitation or animal cruelty because they have their own children or pets. “After being a parent, I am sensitive to child cases and most probably I’ll not be able to hold my emotions if someone speaks to me when I’m working on a child case” (Pt. 35). According to Ehlers and Clarke (2000) model of PTSD, linking personal meanings to a traumatic event, due to one’s own personal experiences, is a key factor in maintaining traumatic symptoms.

Approximately, one-fourth (*n* = 9) of participants felt “miserable” as they could not easily trust people “I’ve lost immediate trust in people so I don’t tell them I work for the Police” (Pt. 34) and as a result were “cynical” while communicating to people outside their work. “At times, you feel miserable that you cannot talk to people about your work as you cannot trust them” (Pt. 18). Some of them (*n* = 16) spoke about “hopelessness” (*n* = 21) in the world and this was linked to the perception that nothing could be done to stop and prosecute criminals because crime would never come to an end. Being hopeless and cynical are key elements of burnout and a loss of trust in others is related to STS (Koutsimani et al. 2019; Lee et al. 2020). However, a couple of them (*n* = 3) felt “gratitude” for what they had and being safe in this unsafe world.

#### Sleep Problems

Finally, around 50% of the participants reported disturbed sleep due to the exposure to distressing content. Some of them elaborated that this disturbance was due to the “recurring thoughts and images.” “I was unable to stop thinking of a case I was working on and those thoughts won’t let me sleep well” (Pt. 15). “Occasionally, images pop into my head and I’m unable to sleep” (Pt. 8). This illustrates that secondary trauma symptoms can lead to poor sleep. In addition, sleep problems have been proposed as both a causal and maintenance factor for PTSD (Biggs et al. 2017; Koffel et al. 2016). In addition, people experiencing sleep problems are at risk of burnout (Metlaine et al. 2018).

Therefore, from the findings, it is evident that these secondary investigators and analysts are at significant risk of developing STS (e.g., uncertainty about the world, loss of trust, hyper vigilance, cognitive avoidance of situations, precautionary behaviors, negative emotions, and recurring thoughts) and burnout (e.g., cynicism, hopelessness, and sleep problems).

#### Coping Strategies

This theme captures the coping strategies that analysts and secondary investigators use to manage the challenging nature of their job. Some of these strategies were used when working with the material while others were used when away from the material. Several coping mechanisms were described by our participants and some of them used more than one strategy to cope with the nature of their work.

#### Coping Strategies when Working with Material

##### Taking Breaks from the Material

Around three-quarters of our participants mentioned “taking breaks” when working with the material. In these breaks, they would step away from their desks and engage in activities like having a warm beverage or talking to their colleagues about something outside of their work.

##### Detachment-Based Strategy

Participants using this coping strategy were detaching themselves emotionally from the content to minimize its impact on their mental health. Approximately two-thirds (*n* = 22) of the participants stated their “level of processing distressing material was shallow.” For instance, these professionals dealt with the material “academically” so that they focus on coding or solving the crime rather than “getting emotionally attached to the victims.” “I focus on the coding or analyzing bit rather than getting emotionally attached” (Pt. 31)*.* Some of them (*n* = 11) analyzed the material like “a puzzle” where they focused on the relevant details to bring parts of information together. They were dealing with the traumatic material as “fiction” or they were treating the “material as unreal,” rather than connecting with it emotionally. Similarly, several of them (*n* = 10) described analyzing the material while wearing an “analytical or investigator hat” so that they could “adopt an analytical focus” to maintain emotional distance.

##### Acceptance

Our participants also mentioned use of acceptance as a mechanism when they “could not do anything to help the victims.” Furthermore, considering the material “as a part of job” and acceptance of good and bad people in the world helped them in dealing with their challenging role. “We’re doing what we can do, I understand, I cannot help everyone” (Pt. 22).

##### Desensitization

Those participants who had considerable experience (*n* = 13) in their role as an analyst and secondary investigator perceived themselves to be “less shocked” because of their “repeated exposure” to the traumatic material.

##### Communication

Another common strategy used by the interviewees was talking to their colleagues and peers, or line managers, about how they feel about a case they were working on. Several of them (*n* = 24) mentioned “chatting with their colleagues” and some of them (*n* = 12) “use humor” to handle the work content that was causing distress. “We laugh about it but not in insensitive way – it is just light (humor), to think about something else” (Pt.31). A few of them (*n* = 8) avoided talking to colleagues about their work initially but with time and more exposure have started to do so.

##### Awareness and Preparedness

Some of the experienced professionals (*n* = 5) mentioned that being forewarned by colleagues about the challenging nature of a case helped them mentally prepare and cope with the case. In this way, awareness acted as a buffer to deal with such cases. “When you’re not mentally prepared for something it affects you more. So, if you prepare the person, when my colleagues prepare me that this is terrible to hear, I’m mentally ready for that case” (Pt. 34).

##### Avoidance of Stimuli

Some interviewees (*n* = 12) “deliberately avoided material” that was similar to their personal experiences. For instance, they avoided children-focused crime cases after having their own children. However, this flexibility was not possible in all units. A few of them (*n* = 8) mentioned that when working with material that had both a visual and audio element, they avoided listening to the audio to reduce the emotional impact.

#### Coping Strategies when Away from Material

##### Compartmentalization

Fifteen of our participants reported that they have “naturally had the ability to cope” with the content of their job and they did not require any coping strategies as they “compartmentalize” their feelings when working with the traumatic material. For example, they consciously did not blend their home and work life*.* “I compartmentalize the information in my head, like, this is work and when I’m going home, I just switch off” (Pt. 5).

##### Thought Suppression

This was a popular strategy employed by participants (*n* = 24) who visualized the material while analyzing it. They actively suppressed the recurring “unwanted thoughts” related to other people’s trauma. “When I [am] alone at home, I think about home intrusion cases … I tell myself it’s of no use thinking of this and I try to suppress that thought” (Pt. 17).

##### Safety Behaviors

As noted above, almost all participants reported engaging in precautionary behaviors as a result of working with traumatic material. For example, more than half (*n* = 19) spoke about “avoiding dark alleys,” “quiet roads” when going back home, and “avoiding watching crime videos or novels” whereas some (*n* = 7) had “stopped running” in late evenings or early mornings when there was not sufficient daylight. Twelve of the interviewees “avoided taking taxis” when coming home late at night. Although these can be interpreted as a negative impact of the exposure to traumatic material, since interviewees are constraining their life experiences, the interviewees saw this as a coping strategy and reported it as a safety behavior

**Support**

##### From Employers

In terms of support from within the organization, our interviewees (*n* = 28) mentioned “availability of supporting resources” on their internal IT systems where there were details of counseling and well-being services. Moreover, their employers were supportive by asking them to take regular breaks while working with the traumatic material and “encouraging them to speak to their colleagues.” “My line manager is supportive and we often get emails from our well-being services as well” (Pt.15). Their managers also provided “flexibility” where possible to them when they were not comfortable with some case content due to their personal experiences (*n* = 13). Some of the participants (*n* = 18) said having psychological assessments every 6 months were helpful and some (*n* = 11) stated that their line manager would be their “first point of contact for support” if they were struggling. “My line manager is really good, she will be my first point of contact for support. She is amazing” (Pt. 22).

##### From Significant Others

Due to the nature of their job, our participants “did not share case contents” with significant others, like their friends and family, due to the sensitivity and confidentiality of the crime incidents on which they were working. However, their significant others, specifically their partners, did provide some support to a few of them (*n* = 11) by “diverting their attention” from work.

##### From Peers

Nevertheless, more than half of them (*n* = 22) mentioned their peers or colleagues as the “best form of support,” most likely because they interact with them on a daily basis at work. Colleagues were always available to them and happy to discuss “sensitive cases” without the feeling of being judged. “When I’m overwhelmed with what I’m reading, I just take a break and chat with my colleagues. These discussions are really useful…” (Pt. 33).

##### Relaxing Activities

Lastly, some participants (*n* = 14) described engaging in activities like “cooking,” “baking,” “listening to music and reading,” “cycling,” “running,” “doing mindfulness,” “yoga,” and “exercise” to cope with the nature of their work.

### Protective Factors

This theme included factors like “Personality” and “Motivators” that participants spoke about in terms of what they felt protected them from being distressed when working with traumatic material on a daily basis.

#### Personality

Some of the interviewees (*n* = 19) mentioned having an “optimistic” personality where they always see the positivity of the tasks they are involved in and this acts as a motivator. For example, they were performing this job to catch the criminals and stop them from doing crimes. “My approach is always positive and optimistic – I think it is my personality to look for positive possible outcome” (Pt. 5). “It’s natural, I get motivated when I see victims – I feel the need to help them” (Pt. 34).

#### Motivators

Our participants revealed a number of motivators that acted as a buffer to the negative impact of the material. Around half of them (*n* = 17) stated that their role was “intellectually stimulating” because they were involved in looking for similar patterns across hundreds of cases and “academically challenging” by identifying offenders’ behavior. Hence, the challenging nature buffered the impact of traumatic material on their mental health. For some of them (*n* = 10), every day was a different day at work because they were dealing with different cases; hence, they enjoyed the “unique and complex” nature of their job. All the interviewees stated that they were motivated because they had a “desire to help people” and make a difference indirectly by detecting suspects and their offending, thereby preventing crime, and identifying strategies to deal with offenders. “It is interesting… I’m here to help people indirectly although I’m not a front-line officer” (Pt. 8). However, for a few of them (*n* = 8), the “nice supporting environment” buffered the effects of working with distressing material. For example, they have opportunity to take breaks and speak to colleagues when overwhelmed with case content and support mechanisms were in place if help was required. Therefore, the supportive working environment was a further factor (Birkeland et al. 2017; Heffren and Hausdorf 2016).

### Additional Risk Factors

Our interviewees mentioned additional risk factors, like “Pressures to Meet Targets” and “Organizational Culture” that influenced their well-being and caused psychological distress.

#### Pressures to Meet Targets

On top of the challenges associated with the distressing material, an additional challenge for many of the participants (*n* = 28) was “meeting deadlines.” They felt that the focus of their managers was on the “quantity rather than the quality” of their work. Related to pressure to meet targets was the feeling that there were insufficient staff to manage the volume of work and quick turnarounds were expected (*n* = 21). “It is just two of us in the office, one is on leave so you can imagine the workload” (Pt. 27). Furthermore, with fewer staff, they were being exposed to more distressing material. Hence, they were at greater risk of burnout. Some indicated that the effects of workload and content were cumulative. “I think the workload is causing burnout for me along with the content but mostly workload, I can’t sleep well due to this” (Pt. 21).

#### Organizational Culture

This sub-theme focused on aspects that were shaping our participants’ perceptions about their employer and organization. For example, the interviewees (*n* = 18) said that there were “no other coping strategies” suggested by their employers for coping with distressing material other than taking breaks and speaking to colleagues. Hence, their employers were “paying lip-service to well-being” that is to say they focused on the work output rather than well-being or there was more talk than action. Some felt the organizations were concerned about their “reputation” rather than staff well-being. “To be honest, I feel it is more talk, when you go for help it is not useful or sufficient I would say.” (Pt*. 33).* They also felt their mental well-being was “neglected by their employers” as greater value was given to frontline staff and when support was provided it was “reactive rather than preventative.” “All the support is reactive there is nothing preventative for us” (Pt. 15). In addition, one-fourth (*n* = 8) stated that the supporting resources were “not meeting their needs” as they were not well-equipped, were not meeting required standards, or were simply unavailable. In some cases, previous resources for well-being had been withdrawn. They put this down to economic pressures on the organizations. “We used to have [a] psychologist but we don’t have her anymore because we don’t have enough budget maybe?” (Pt. 24).

Some (*n* = 20) referred to psychological assessments that were conducted every 6 months to check if the employees were fit to perform this challenging job. However, they felt that assessments were not fit for purpose as they did not focus on *improving* their mental health and well-being and instead were about ensuring they were sufficiently “fit.” Participants observed that it was easy to “provide desirable responses” to the assessment questions to stay in the job.

Participants perceived their “management as having limited knowledge” of their role and hence their decisions regarding well-being were not well-informed. “Management have their own agenda and are interested in moving up in their roles” (Pt. 35). Moreover, not all line managers kept a check on their immediate employees’ mental health, rather they focused on the set targets. “I’ve not heard from my managers on my well-being, it is more about you have not met targets and what could be the reasons for it” (Pt. 5). A number of participants (*n* = 23) mentioned about inadequate provision of well-being services. Explicitly, there was “no specific training” for dealing with the traumatic material. “We have been not given any training, if you have no police background it is difficult to know what you will come across in this role” (Pt. 35). “Those who are uniformed officers had training on how to deal with this content but we as civilians have not been given any training” (Pt. 20). This made them (*n* = 14) feel their employer was treating them unfairly; while they were not trained on how to deal with the material and the organization did not provide any financial support for seeking private therapies, these were provided to uniformed employees. In addition, a couple of them (*n* = 6) mentioned “lack of career development” and several of them (*n* = 15) mentioned “lack of confidentiality.” For example, mental health problems were still considered a stigma where if employees ask for help from their employers, they were considered incapable and weak in performing their job appropriately and this would have consequences for career progression.

#### Consequences Due to Additional Risk Factors

Pressure to meet targets and workload led to physical and mental exhaustion and sleep problems. “I get nightmares about my work, aspects like not meeting deadlines…” (Pt. 34). “At first, I was not taking breaks and working extra hours to meet the deadlines but that was exhausting” (Pt. 32). Others (*n* = 5), who lived on their own, reported feeling isolated and working overtime to meet monthly targets. This was leading to intrusive thoughts, poor sleep, and decreased productivity. “Due to the consistent workload, I can’t stop thinking of negative images whereas before I was able to cope well” (Pt.33). Even though participants reported being keen to seek help from well-being services, they also reported having insufficient time to do so due to their workload. For a few of them (*n* = 3), the organizational culture of supporting colleagues with caring responsibilities was having a knock-on negative effect. By having no family/children, they were “expected to work more hours” and could not take leave as easily. “I’m happy that I have children now, as first when I used to take leave, my colleagues would be not happy with me … who is going to cover me” (Pt. 35). In the published literature, physical and mental exhaustion due to work burden is considered signs of burnout that are related with negative thoughts and psychological distress (Chang et al. 2017).

In addition, ten of them had “intentions to leave their job” if they had a better option and one of them had switched roles. Employees whose managers were not supportive in providing mental health support preferred to seek private support rather than approaching their employer when the work was impacting their psychological well-being. “I will seek private support when required due to the stigma, and they (detectives) will also think I’m not capable of doing my job” (Pt. 26).

## Discussion

The main aim of this study was to explore the experiences of analysts and secondary investigators working with other people’s trauma indirectly on a daily basis in back-office roles in police, law enforcement, and justice organizations. Furthermore, we aimed to understand the coping mechanisms they practice to manage the challenging nature of their job.

From our interviews, it was evident that these analysts and secondary investigators were exposed to a wide range of traumatic material on a day-to-day basis in different forms. This exposure was acting as a work stressor and affecting their mental health (Brady 2017; Cieslak et al. 2007; Lee et al. 2020). Nevertheless, these interviewees were motivated because of their optimistic personality and their desire to help people that gave them a sense of pleasure and satisfaction from their work. However, it is worth noting that it is difficult for these professionals to help others unless they are psychologically fit themselves (Brady 2017). The mode through which they received the information about trauma and the ways they analyzed it differed across these professionals. Information about other people’s traumatic experiences was received by them via auditory and visual means, or solely visual, or solely auditory. When working with the material, they reported adopting different perspectives (e.g., taking the perspective of the victim or the perpetrator).

Our participants reported a range of negative reactions to the traumatic material as a consequence of constant exposure, including secondary traumatic stress and burnout symptoms (Bride et al. 2007; Perez et al. 2010), intrusive thoughts, avoidance behavior, hyper vigilance, suspiciousness, cynicism, emotional and physical exhaustion, sleep problems, and excessive precautionary behavior.

Constant exposure to distressing content was negatively influencing participants’ thoughts and feelings about the world, home, and social life. For example, some perceived danger to be everywhere with crime being associated with a range of situations and environments (e.g., home, in the street, in other people’s homes). Following on from this, some participants felt a sense of hopelessness. In some instances, this was associated with the low likelihood of criminals being successfully prosecuted. Receiving feedback when there has been a successful prosecution could, therefore, be very important and it might give them a sense of achievement (Lee et al. 2020) and affirm their motivation for continuing in their work (i.e., to help others). Nevertheless, experienced analysts and secondary investigators were desensitized to the traumatic content due to repeated exposure; perhaps, this might be because they have developed resilience due to the continuous exposure with time (Richardson 2002).

According to models, such as Ehlers and Clark (2000) model of PTSD, some of the impacts reported by participants (intrusive and negative thoughts, hyper vigilance, etc.), as well as some of the coping mechanisms reported, are problematic in *maintaining* a PTSD-type reaction to their work. For example, avoidance behavior and adopting excessive precautionary behavior prevent the individual from learning that being in the same circumstances as a victim does not always lead to a crime being committed. Hence, the sense of current and global threat that some are experiencing is not challenged or diminished.

In addition to ongoing exposure to distressing material, additional risk factors were reported as causing psychological distress. High caseloads with time-related targets and fewer staff, as well as feeling under-valued, were causing mental and physical exhaustion and sleep problems. This is consistent with previous literature (Lewis et al. 2013; Stevenson 2007) that exposure to traumatic material alongside high caseloads is related to burnout. STS and burnout symptoms were clearly contributing to these professionals’ decisions to leave as per previous research (Kinzel and Nanson 2000; Perez et al. 2010). These findings highlight the importance of adequately staffing units where personnel hold high-risk roles and ensuring that uniformed *and* civilian staff are treated fairly and are valued within the organization. They also suggest that managers of personnel in high-risk roles should avoid time-related target setting.

While some participants spoke positively about the support received from colleagues and supervisors, the approach of some organizations was criticized for being reactive rather than preventative, with coverage in training of preventative measures being particularly limited. Inadequate training on how to deal with trauma content has been noted by others (e.g., Lee et al. 2020). Problematic cultures where asking for support was stigmatizing were reported. Existing studies have linked mental health stigma in the workplace with isolation, stress, lack of communication, and low mood (Papazogolu 2013). Hence, cultivating a workplace culture where mental health is openly discussed and is a priority is a key, and efforts are already being made in policing and law enforcement to address this (for e.g., see the work by Oscar Kilo and the Blue Light Programme).

In addition to support provided by their employers, the participants had themselves developed their own coping strategies: taking short breaks from the material, chatting with their colleagues, and partaking in activities like exercising, cooking, doing yoga, and practicing mindfulness. Some reported being able to compartmentalize work and home thereby protecting themselves as well as shielding their families from their work. In contrast with previous findings (Bornstein et al. 2005; Lepore 2001), support from significant others like a partner, family members, and friends was not perceived as being as useful because the sensitive and confidential nature of their work meant they could not share work content with such parties. Instead, they sought support from their work colleagues when dealing with traumatic material to enhance cognitive and emotional processing of trauma-related thoughts and feelings (Lepore 2001; Vrklevski and Franklin 2008). Having the opportunity to seek support from colleagues is therefore important and, conversely, barriers to this (e.g., insufficient time, colleagues not co-located) could cause distress (Lepore 2001).

Some participants volunteered coping strategies that could be maladaptive. For example, some used thought suppression to cope and models of PTSD (e.g., Ehlers and Clarke 2000) caution that such methods could exacerbate PTSD-like symptoms such as intrusive thoughts (Birrer et al. 2007; Miller et al. 2019). Similarly, emotional detachment and processing material at a shallow level could aggravate PTSD-like symptoms as they prevent sufficient processing of the traumatic material to avoid or escape the fear (Shay and Munroe 1999). As noted above, avoidance and excessive precautionary behavior can also have adverse effects and were also cited by participants as coping strategies that they adopt.

In our study, we did not rely on the common categorization of coping mechanisms into problem-focused and emotion-focused coping due to criticisms of these categories for being over-simplistic if trying to identify important sub-dimensions in coping (Biggs et al. 2017). However, our findings fit with the Transactional Theory of Stress and Coping (Lazarus and Folkman 1984) where stress initiates coping behavior to (a) manage emotions (emotion-focused) or (b) address the stressor (problem-focused). For example, our participants articulated examples of emotion-focused strategies (such as acceptance, humor, support, avoidance). However, other examples mentioned by them are not easy to be categorized, for example, taking breaks when working with the material could be considered a problem-focused strategy as an attempt to address a stressor; however, our participants also mentioned it as a strategy to cope with overwhelming emotions.

How these various factors can coalesce to negatively affect the well-being of analysts and secondary investigators can be explained using the Job-Demands Resource model (JD-R; Bakker and Demerouti 2007). According to this model, resources (physical, psychological, social, or organizational) assist an individual in reaching work goals, stimulate growth and development, and reduce job demands and subsequent strain felt by the individual (Demerouti et al. 2001). Thus, strain is the detrimental effect of a mismatch between demands (physical, psychological, social, or organizational) and resources whereby high job demands *and* insufficient resources negatively influence mental health and well-being (Bakker and Demerouti 2007). In this study, high job demands have been reported: working with traumatic content on a regular basis, a high caseload, and being subject to time-related targets. Alongside these, the following evidence for insufficient resources has been cited: reduced staff numbers, inadequate training and support services, and a stigmatizing culture where confidentiality is not respected. Therefore, due to the nature of the material, the demand of working with it could not be addressed; however, by giving staff breaks, fewer time-targeted benchmarks and deadlines, and providing them flexibility to work on cases they are comfortable with could decrease the demands on them. Moreover, employers could boost or remove the barriers to resources by providing adequate training, hiring more staff so there is less exposure to traumatic experiences of other people, ensuring confidentiality by building a trusting relationship, and encouraging employees to speak to their managers when required. These strategies might be useful in addressing the STS and burnout symptoms. In addition, psychoeducation could be another useful resource provided by managers to educate their team members on the risk of burnout and STS via regular staff or debrief meetings (Starcher et al. 2021). Explicitly, it would involve a cluster of activities about increasing awareness and supportive supervision to build resilience and organizational policies to monitor STS (Molnar et al. 2017; Sprang et al. 2017).

Moreover, organizations could create a culture where mental well-being is valued. For example, one of the organizations mentioned having monthly well-being seminars where all analysts shared their experiences of working with traumatic material to reduce psychological distress. Hence, similar approach could be adopted by other organizations but they need to make sure that work demands allow analysts and secondary investigators to take up these opportunities (Chanana and Sangeeta 2020). Thus, our findings provide an opportunity for employers to design healthy interventions and organizational culture that effectively reduce burnout and secondary trauma symptoms to safeguard their employees’ psychological well-being (Perron and Hiltz 2006). Therefore, this implication could be successfully projected by arranging workshops with multiple stakeholders, subject experts, well-being representatives, clinical psychologists, and policymakers to discuss (Lee et al. 2020) and co-produce recommendations for workplace interventions to prevent the deleterious effects of exposure to distressing material through work on their mental health.

This group of professionals are neglected group by academics; hence, this is the first international study exploring the experiences of secondary investigative and analytical staff who work as supportive staff in different back-office departments/units. However, we acknowledge several limitations to the work. First, the respondents self-selected to participate because they wanted to talk about their mental well-being and felt comfortable sharing their experiences with the researcher. Second, this research was conducted within the COVID-19 pandemic; therefore it might have impacted their responses when working with traumatic content (see Duran and Woodhams 2022). Third, although the data were rich and were collected from different organizations within the UK and internationally, there might be some factors that relate more to one organization than others and this could be addressed by a case-by-case analysis. Following on from the ethical approval for our study, it was not possible for us to investigate this without compromising the anonymity of our participants and the organizations for whom they work. Fourth, to generalize our findings, this work could be extended to other countries that have not participated in this study. Finally, this study was cross-sectional in nature and there is a possibility that the experiences of these professionals change with time. Indeed, some, more experienced, participants themselves reflected on the fact that their reactions to the material had changed with time. A longitudinal, mixed-methods study would provide evidence for such changes and offer an insight into the causal mechanisms responsible for the onset and maintenance of mental health problems, specifically STS and burnout.

## Conclusion

In summary, analysts and secondary investigators play an important role in protecting our societies and ensuring justice for victims, yet they are at risk of developing secondary traumatic stress and burnout as a result of ongoing exposure to traumatic material that is a necessary part of the work they conduct. Our findings provide an in-depth understanding of why and how the work these professionals are engaged in could cause harm to their mental health, as well as the strategies they and their employers could adopt to maintain their mental health and well-being while in these important roles. Our findings are also relevant to professionals in related roles whose work brings them into regular contact with traumatic material (e.g., police and court translators, solicitors, charity workers, and social media moderators).
